# Study of Anticorrosion and Antifouling Properties of a Cu-Doped TiO_2_ Coating Fabricated via Micro-Arc Oxidation

**DOI:** 10.3390/ma17010217

**Published:** 2023-12-30

**Authors:** Pengfei Hu, Liyang Zhu, Chenghuan Tian, Gege Xu, Xinxin Zhang, Guangyi Cai

**Affiliations:** 1 National Key Laboratory of Electromagnetic Energy, Naval University of Engineering, Wuhan 430033, China; 2 East Lake Laboratory, Wuhan 420202, China; 3 Hubei Key Laboratory of Material Chemistry and Service Failure, School of Chemistry and Chemical Engineering, Huazhong University of Science and Technology, Wuhan 430074, China

**Keywords:** micro-arc oxidation, titanium, Cu incorporation, antifouling, anticorrosion

## Abstract

As a promising material for petroleum industrial applications, titanium (Ti) and its alloys receive wide attention due to their outstanding physicochemical properties. However, the harsh industrial environment requires an antifouling surface with a desired corrosion resistance for Ti and its alloys. In order to achieve the desired antifouling properties, micro-arc oxidation (MAO) was used to prepare a Cu-doped TiO_2_ coating. The microstructure of the Cu-doped TiO_2_ coating was investigated by TF-XRD, SEM, and other characterization techniques, and its antifouling and anticorrosion properties were also tested. The results show the effects of the incorporation of Cu (~1.73 wt.%) into TiO_2_ to form a Cu-doped TiO_2_, namely, a Ti–Cu coating. The porosity (~4.8%) and average pore size (~0.42 μm) of the Ti–Cu coating are smaller than the porosity (~5.6%) and average pore size (~0.66 μm) of Ti–blank coating. In addition, there is a significant reduction in the amount of SRB adhesion on the Ti–Cu coating compared to the Ti–blank coating under the same conditions, while there is little difference in corrosion resistance between the two coatings. There, the addition of copper helps to improve the fouling resistance of TiO_2_ coatings without compromising their corrosion resistance. Our work provides a practical method to improve the antifouling function of metallic Ti substrates, which could promote the application of Ti in the petroleum industry.

## 1. Introduction

In recent years, as the exploration of crude oil has gradually moved to deep water, conventional oilfield pipes have had difficulties in meeting the requirements of the increasingly harsh environment [1,2,3]. Ti and its alloys have been applied to drilling platforms in pipelines in the petroleum industry due to their excellent physicochemical properties and high corrosion resistance [4,5].

Although Ti and its alloys usually exhibit excellent corrosion resistance due to the passive film on their surfaces, the presence of microorganisms may damage the passive film and cause corrosion of the Ti [6,7,8,9]. Among the most widely existing microorganisms in crude oil are sulphate-reducing bacteria (SRB) [10,11]. Under anaerobic conditions, SRB derive energy from the reoxidation–reduction reaction, which uses hydrogen produced in the bacterial biofilm to reduce sulphate to hydrogen sulphide. Then, the pH of the local chemical environment will decrease, which will destroy the stability of passive film and enhance the corrosion sensitivity of metal [10,12]. Microbiologically influenced corrosion (MIC) may occur when the SRB biofilm is formed because hydrogenase from the SRB biofilm promotes a cathodic reaction, and the anodic dissolution of metal will be accelerated [13,14,15,16]. In addition, the extracellular polymeric substance (EPS) generated from SRB biofilm could combine with metal ions, which may also accelerate the corrosion of the metal substrate [17]. Once formed, SRB biofilm is extremely difficult to remove completely as it prevents the penetration of antimicrobial agents into the biofilm [18]. Therefore, the prevention of SRB biofilm formation on the metallic Ti substrate surface could be an effective method to avoid MIC damage, which requires the enhancement of antifouling capabilities against SRB on the Ti surface. Among the various corrosion control techniques available, protective coatings, inhibitors, anticorrosive strains, and modification of the surface by attaching anticorrosive functional groups on the surface are commonly used [19,20,21,22].

Cu has been widely used as an effective antimicrobial agent to inhibit bacterial growth and prevent biofilm formation [23,24]. Therefore, it is foreseeable that the incorporation of Cu will improve the antifouling effect of the Ti surface. Among all viable surface modification methods, micro-arc oxidation (MAO) is one of the most effective methods to fabricate a metallurgically bonded Cu-doped coating on a Ti substrate [25,26]. MAO uses a high external voltage to promote a plasma-assisted anodic oxidation process on the metal surface, which could form a ceramic oxide layer with the desired hardness and chemical inertness [27,28]. At the coating/substrate interface formed by MAO, there is a metallurgical bond, and the ceramic coating is inert. Therefore, the wear and corrosion resistance of the Ti substrate could be enhanced by MAO [29,30,31,32]. The effective method for forming coatings is plasma electrolytic oxidation (PEO) in aqueous electrolytes [33] and molten salt [34]. The chemical composition of the ceramic coating can be easily adjusted by changing the electrolytic composition of the MAO. In order to incorporate Cu into TiO_2_, Cu-containing components could be added to the MAO electrolyte [35]. Alternatively, a Cu-rich substrate could also be used as the anode in the MAO process [36]. Since most Ti alloys are Cu-free, Cu-containing electrolyte components are commonly used as Cu sources for incorporation into TiO_2_. Unlike the traditional use of Cu salts and Cu-rich particles [37], in our previous work, ethylene diamine tetraacetic acid cupric disodium (Na_2_Cu–EDTA) was added in the electrolyte as a Cu source for the MAO treatment [38], which can take advantage of the external electric field to promote Cu incorporation. However, studies on the antifouling ability of Cu-doped TiO_2_ coatings prepared using Cu–EDTA complexes are still limited.

In this work, instead of traditional Cu sources, Na_2_Cu–EDTA was added to the electrolyte as a Cu source for the MAO treatment. During the MAO process, the electric field promotes the mass transfer of negatively charged Cu–EDTA complexes to the Ti substrate, which favors the incorporation of Cu into the TiO_2_ coating. The addition of Na_2_Cu–EDTA results in a less defective appearance of the ceramic oxide. Therefore, Na_2_Cu–EDTA is a promising Cu source for the fabrication of Cu-incorporated TiO_2_ coatings. The corrosion and fouling resistance of the Cu-doped coatings was investigated by comparison with Cu-free TiO2 coatings and metallic Ti substrates. Antifouling capability is enhanced after Cu incorporation. These results could offer some theoretical guidance to promote the application of titanium and its alloys in the petroleum industry.

## 2. Materials and Methods

### 2.1. Preparation of Specimens

**Figure 1:**
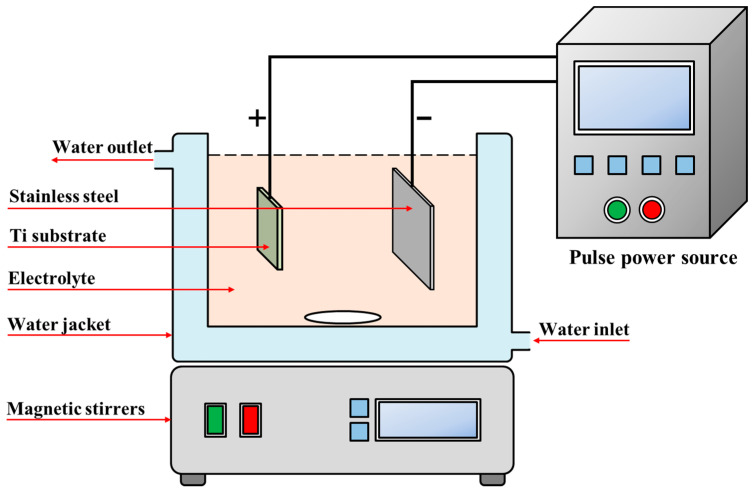
Schematic diagram of MAO process.

The specimens were commercially pure titanium (cp-Ti) and were polished to 1200 grit, washed with high purity water, and finally dried in a cool air stream. The MAO treatment was carried out on cp-Ti samples using the apparatus (YS9000DD, Yisheng, Shenzhen, China) shown in Figure 1. During the MAO process, the galvanostatic mode was used, its current density, frequency, and duty cycle optimized at 80 A·cm^−2^, 600 Hz, and 10%, respectively.

Two electrolytes were prepared for the MAO process. By gradually dissolving 15.8 g calcium acetate and 7.2 g sodium dihydrogen phosphate into 1 L water, the electrolyte was prepared to fabricate a Cu-free TiO_2_ coating, which was named as Ti–blank in this context. Further, an additional 5 g Na_2_Cu–EDTA was added to the above electrolyte, and the corresponding coating named as Ti–Cu coating. After the MAO treatments, the specimens were cleaned by deionized water and dried in a cool air stream before further examination.

### 2.2. Characterization

TF-XRD (thin-film X-ray diffraction, XtaLAB PRO MM007HF, Rigaku, Tokyo, Japan) was used to detect the phase component of the MAO specimens. At the incident angle of 0.8°, it was swept from 20° to 80° at a speed of 1° min^−1^. The surface and cross-section morphologies of samples were examined using a field emission scanning electron microscope (SEM, Gemini 300, Carl Zeiss AG, Jena, Germany). Water contact angles (WCAs) of both coatings were measured using an optical contact angle instrument (JC2000D, Xi’an, China). Finally, the surface topographies of both coatings were examined by using a Bruker Contour GT K 3D (Billerica, MA, USA) high-resolution surface measurement system to obtain the surface roughness R_a_.

The traditional three-electrode system is used for the electrochemical test (CorrTest, Wuhan, China) in a 3.5 wt.% NaCl solution; the working area of the sample is 1 cm^2^. In the stable open circuit potential (OCP), the potentiodynamic polarization is scanned from −200 mV (vs. OCP) to +200 mV (vs. OCP) at a rate of 0.5 mV/s. The electrochemical impedance spectrum (EIS) is also measured under OCP, and the frequency range is 10^5^ to 0.01 Hz.

Separated from the Shengli Oilfield (Dongying, Shandong Province, China), the SRB seeds were identified as *Desulfotomaculum nigrificans*. The SRB were cultivated in a culture medium containing 0.01 g/L K_2_HPO_4_, 0.2 g/L MgSO_4_, 0.2 g/L (NH4)_2_Fe (SO_4_)_2_, 10 g/L NaCl, 1 g/L yeast extract, 0.1 g/L vitamin C, and 4 g/L sodium lactate, with its pH adjusted to 7.0–7.2 with NaOH. These chemicals were supplied by Sinopharm, Shanghai, China. The culture medium was sterilized in an autoclave for 20 min at 121 °C, and the specimens were sanitized under a 30 W ultraviolet light bulb for 30 min. To remove dissolved oxygen, the culture medium was then sparged with CO_2_ for 4 h.

After 4 days of incubation of the SRB at 37 °C, all the specimens were then gently rinsed using phosphate buffer solution (PBS, Macklin, Shanghai, China). After that, the specimens were placed in a 2.5% (*v*/*v*) glutaraldehyde for 8 hours and then dehydrated using a series of ethanol solutions (20%, 50%, 70%, 90%, 99% by volume) prior to the examination using SEM. All the experiments were repeated at least three times.

## 3. Results

**Figure 2:**
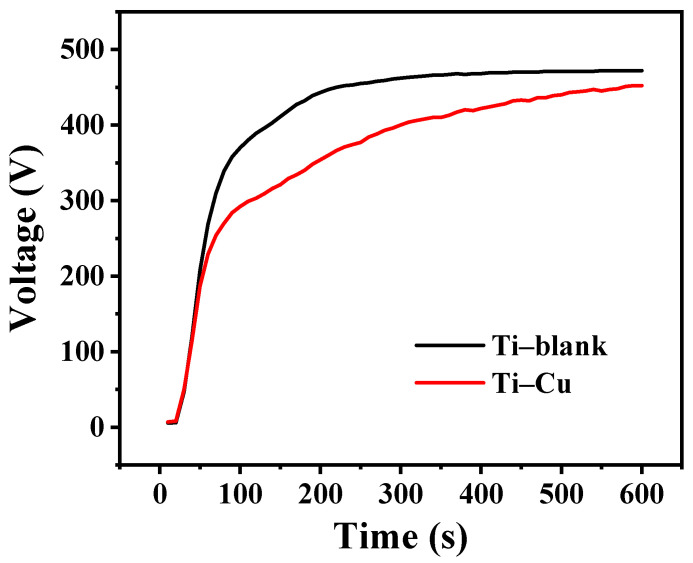
The voltage–time curve of Ti in the MAO process.

The voltage–time curve of Ti in the MAO process of Ti is shown in Figure 2, and the typical cell voltage evolution during MAO can be seen. Initially, the cell voltage increases rapidly, then the rate of increase slows down. Finally, the cell voltage stabilizes with slight fluctuations. It can be seen from Figure 2 that although the trends are similar, the breakdown and stabilization potentials of the Ti–blank coatings (~370 V and ~470 V) are significantly higher than those of the Ti–Cu coatings (~280 V and ~450 V), which may be related to the presence of Na_2_Cu–EDTA in the solutions discussed in the next section.

**Figure 3:**
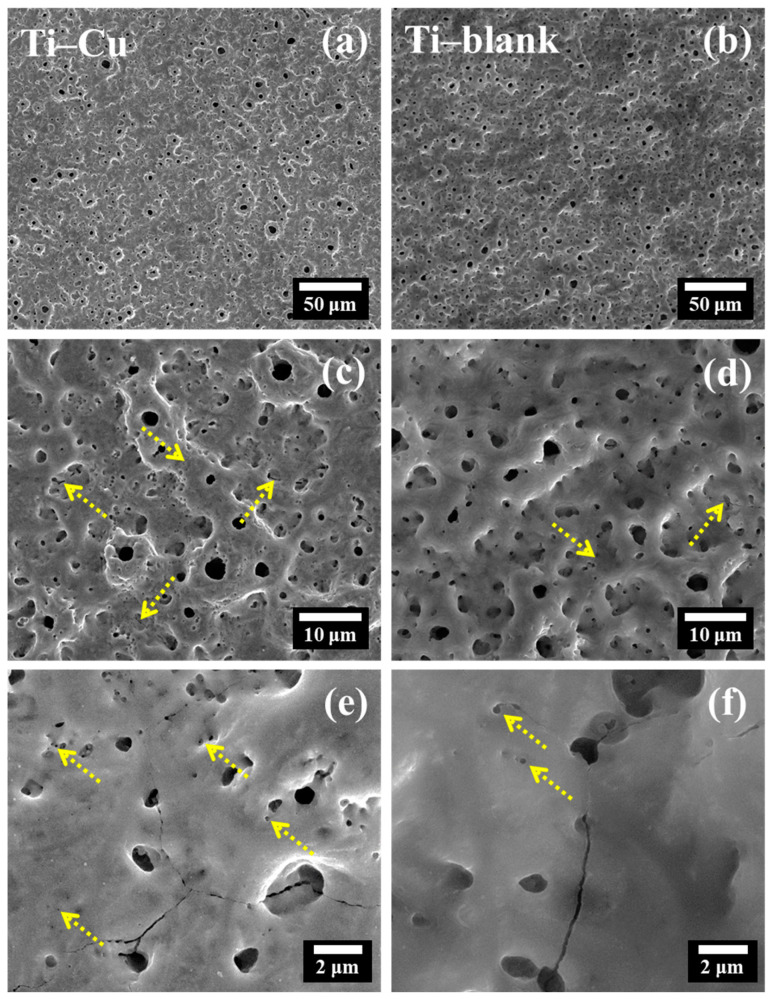
The surface microstructure of Ti–Cu coating (**a**,**c**,**e**) and Ti–blank coating (**b**,**d**,**f**).

**Figure 4:**
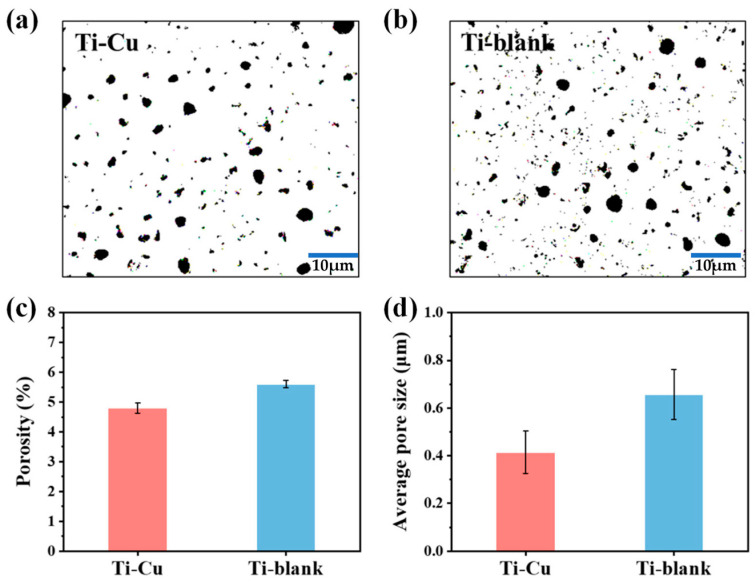
Pore distribution (**a**,**b**), porosity (**c**) and average pore size (**d**) of Ti–Cu and Ti–blank coatings.

Figure 3 shows the microstructure of Ti–blank and Ti–Cu coatings, both of which exhibit the typical porous structure of the MAO process. Figure 3c,d display the magnified views of two coatings, micron-sized and submicron-sized pores attributed to micro-arc discharge events [39]. Porosity reflects the area fraction of micron-sized and submicron-sized pores on the entire coating area, which could be determined by the image segmentation method. Figure 4a,b are the images of Figure 3c,d processed by Image-Pro Plus 6.0 software, and they reflect the size and porosity of the pores. As shown in Figure 4c, the porosity of the Ti–Cu coating (~4.8%) is lower than that of the Ti–blank coating (~5.6%). In addition, the average pore size can be obtained by measuring more than 100 pores, and Figure 4d shows that the Ti–Cu coating (~0.42 μm) has a lower average pore size compared to the Ti–blank coating (~0.66 μm).

Figure 3c,d also reveal a reduced number of cracks in the Ti–Cu coating compared to the Ti–blank coating, with typical cracks marked by arrows, which is attributed to the buffering effect of the Cu–EDTA complex [40], as illustrated later. Finally, Figure 3e,f reveal the presence of cracks and nano-sized pores (marked by arrows) in both coatings. The formation of cracks is originated from thermal stress associated with the sudden change in local temperature, while the oxygen evolution may result in the formation of nano-sized pores in the ceramic oxide [41].

**Figure 5:**
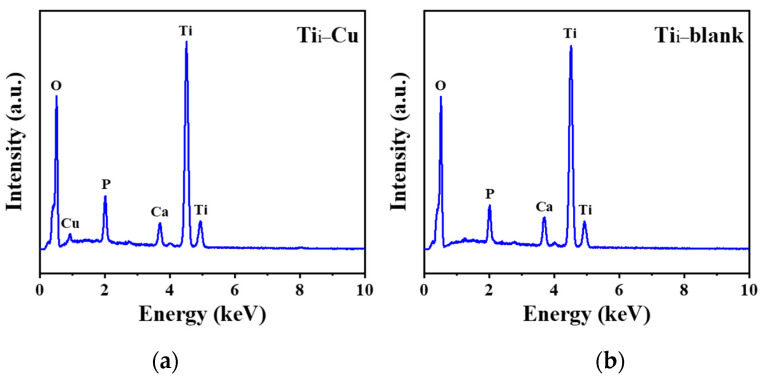
EDX spectra of Ti–Cu (**a**) and Ti–blank (**b**) coatings.

EDX point analysis was carried out to identify the elemental composition of the two coatings, and representative EDX spectra are shown in Figure 5. As expected, Ti, O, Ca, and P were detected by EDX in the Ti–blank coating and Ti–Cu coatings (Figure 5a). In addition, the presence of a characteristic peak for Cu is shown in the EDX spectrum of the Ti–Cu coating (Figure 5b), indicating that the addition of Na_2_Cu–EDTA in the solution has successfully incorporated Cu into TiO_2_. Table 1 shows the quantitative elemental compositions of the two coatings, which confirms the incorporation of Cu (~1.73 wt.%) into TiO_2_ to form a Cu-doped TiO_2_, namely, the Ti–Cu coating.

**Figure 6:**
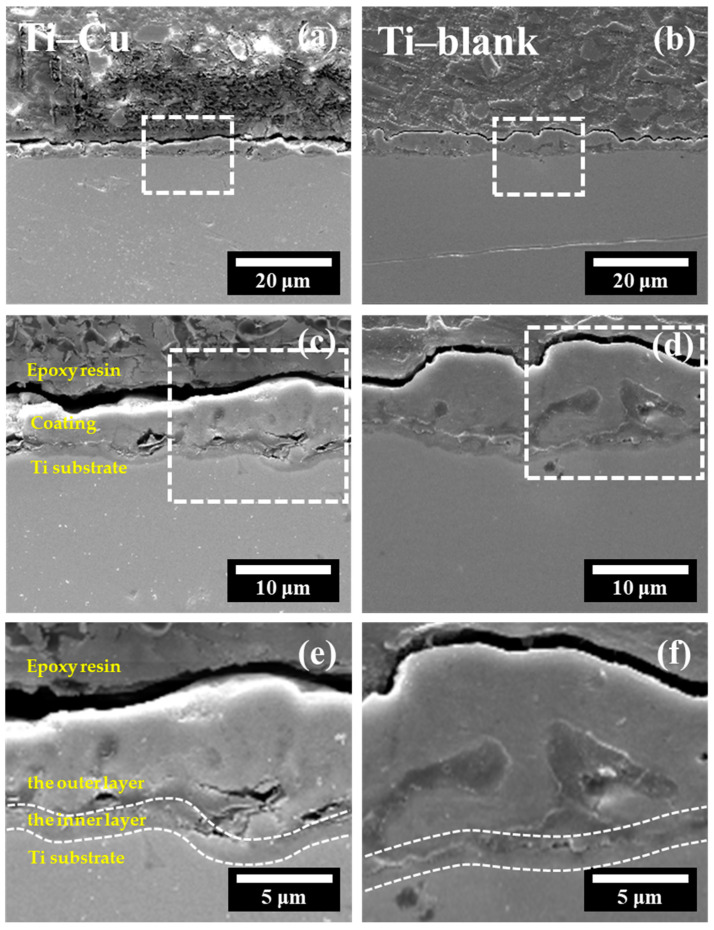
Microstructure of cross-section views in coatings along with EDX line-scan: (**a**,**c**,**e**) Ti–Cu coating; (**b**,**d**,**f**) Ti–blank coating, the dashed squares represents the enlarged area.

Next, Figure 6 shows the cross-sections of Ti–blank and Ti–Cu coatings. The thickness of the Ti–Cu coating ranged from 3.5 μm to 10.8 μm, which is slightly lower than the Ti–blank coating with a thickness range of 3.8 μm to 12.7 μm. Figure 6c,d exhibit the morphological features across the ceramic coatings with porous structures. Figure 6e,f show that these two coatings display bi-layered structures. It can be assumed that the inner layer has a compact appearance, while the outer layer is composed of numerous pores, which is consistent with previous work [42].

**Figure 7:**
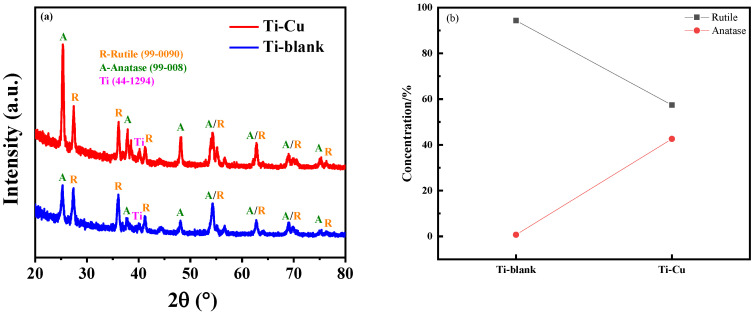
TF-XRD patterns (**a**) and concentration (%) of rutile and anatase phases (**b**) of Ti–Cu and Ti–blank coatings.

Thin-film XRD (TF-XRD) was applied to determine the phase components of the Ti–Cu and Ti–blank coatings (Figure 7a). Clearly, characteristic peaks for anatase TiO_2_, rutile TiO_2_, and metallic Ti were detected in both coatings. The detection of Ti may be related to the low thickness of the ceramic coating (Figure 6), which results in the penetration of X-rays to the Ti substrate. In addition, although rutile and anatase types were detected in both coatings, their relative contents were significantly different. The proportions of anatase and rutile detected by the XRD were assessed by the following Equation [3]:

(1) $$W_{R}=1/(1+0.8I_{A}/I_{R})$$

The corresponding results were shown in Figure 7b. The rutile/anatase ratio was lower in the Ti–Cu layer (~0.574) than in the Ti–blank layer (~0.943), which is related to the micro-arc discharge event, as will be shown later.

**Figure 8:**
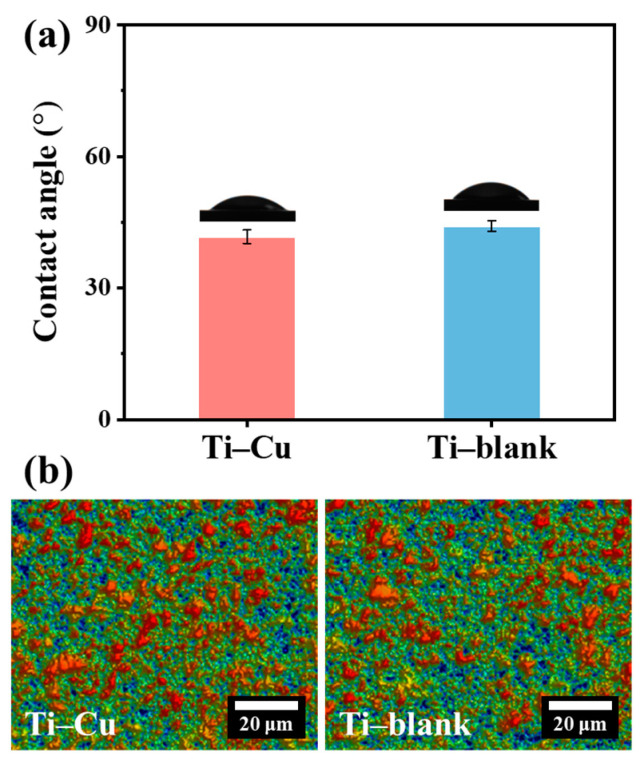
Water contact angles (**a**) and surface topography (**b**) of Ti–Cu and Ti–blank coatings.

After the microstructural characterization, both surface wettability and surface roughness R_a_ were examined with the representative water contact angles and surface topographies shown in Figure 8. The WCA (~41.7°) and roughness Ra (~0.98 μm) of the Ti–Cu coatings were similar to the Ti–blank coatings (WCA~44.1°, Ra~0.95 μm), suggesting that the surface state of the ceramic coatings remained insensitive to the addition of Cu.

**Figure 9:**
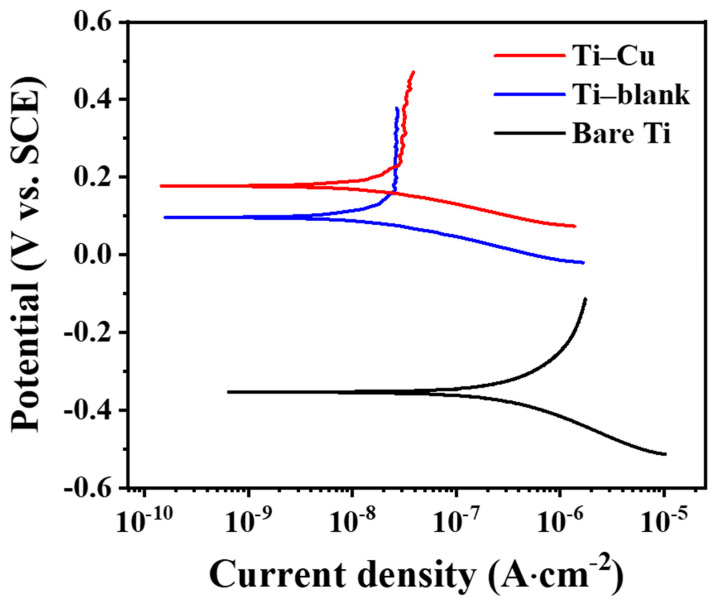
Potentiodynamic polarization curves of Ti–Cu, Ti–blank coatings, and bare Ti.

In order to evaluate the corrosion resistance of the two coatings, electrochemical measurements were performed. Electrochemical measurements were also carried out on a bare titanium substrate for comparison. Figure 9 shows the potential polarization curves, and Table 2 shows the fitting results. The corrosion potential (*E_corr_*) increased significantly, and the corrosion current density (*i_corr_*) decreased after the MAO process. The order of *i_corr_* is that the Ti–Cu coating (1.65 × 10^−8^ A/cm^2^) is approximately equal to the Ti–blank coating (1.64 × 10^−8^ A/cm^2^) and greater than bare Ti (2.1 × 10^−7^ A/cm^2^). Since *i_corr_* is a critical parameter of the corrosion resistance for the specimen, it is preliminarily demonstrated that the corrosion resistance of Ti can be enhanced by the MAO process, regardless of whether Cu is incorporated or not.

**Figure 10:**
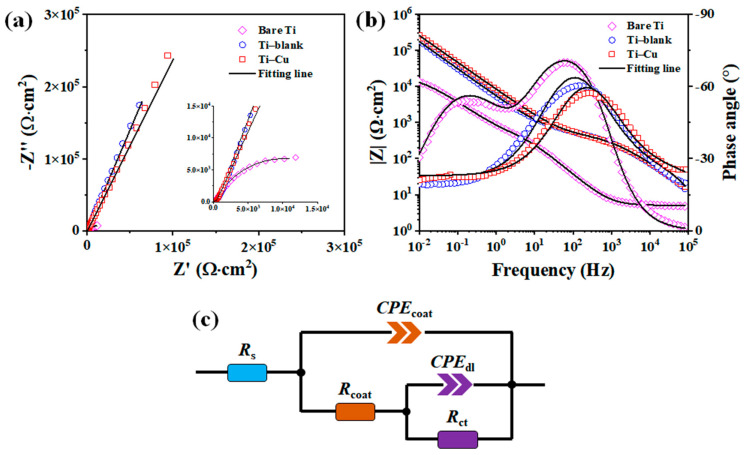
EIS analysis of Ti–Cu and Ti–blank coatings and bare Ti: (**a**) Nyquist plots; (**b**) Bode plots; (**c**) the equivalent circuit (EC).

The electrochemical impedance spectrum (EIS) analysis of these two coatings is shown in Figure 10. The Bode plots (Figure 10b) show that the impedance modulus (|Z|) at 10 mHz is in the order indicating that the Ti–Cu coating is approximately equal to the Ti–blank coating, and both are larger than with the bare Ti. Since the |Z|_10 mHz_ could reflect the corrosion resistance of the examined system, the corrosion resistance of Ti could be improved by the MAO process. Based on the Nyquist plots (Figure 10a), the equivalent circuit (EC) model in Figure 10c is applied to fit the EIS data with the detailed description reported previously [43], where Rs, CPE_dl_, and R_ct_ represent the solution resistance, double-layer capacitance, and charge transfer resistance, respectively. For bare titanium, CPE_1_ and R_1_ represent the capacitance and resistance of the passive film, respectively. For Ti–Cu and Ti–blank coatings, CPE1 and R_1_ represent the capacitance and resistance of the MAO layer, respectively [33]. Table 3 lists the fitted data, where n represents the surface smoothness of the CPE. As the polarization process reaches the low-frequency region, the passive film produced on the electrode surface and the oxide film formed by micro-arc oxidation are rough, and the electrochemical process is controlled by mass transfer, thus resulting in the low n values. The sum of the resistances indicates that the corrosion resistance of the Ti–Cu coating is comparable to that of the Ti–blank coating, while the bare Ti has the worst corrosion resistance. The results again indicate that the presence of MAO coating improves the corrosion resistance of Ti substrates, and the corrosion resistance of MAO coating is insensitive to Cu incorporation. Therefore, according to Figure 9 and Figure 10, it can be seen that the MAO process could effectively enhance the corrosion resistance of the metallic substrate and the incorporation of Cu into an MAO coating remains harmless to its corrosion resistance.

**Figure 11:**
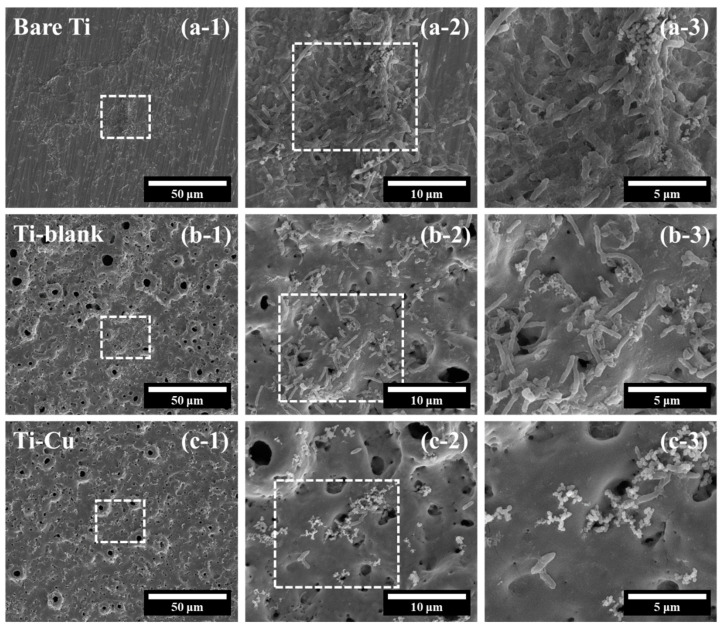
Morphological characterization of SRB on the surfaces of (**a**) Bare Ti, (**b**) Ti–blank, and (**c**) Ti–Cu coatings, the dashed squares represents the enlarged area.

Finally, the antifouling performance of the Ti–Cu and Ti–blank coatings on SRB was investigated and compared with bare Ti. The surface morphology of all samples was examined by SEM after 4 days of incubation. Figure 11a shows the surface views of bare Ti. The general view (Figure 11a-1) indicates the existence of an additional layer, presumed to be a biofilm on the Ti substrate. In order to observe more detailed morphological features, the surface of bare Ti is shown at an increased magnification (Figure 11a-2). The figure shows a number of rod-shaped features, namely SRB bacteria, with their typical dimensions ranging from several micrometers to over 10 μm. Figure 11a-3 shows the fusion of these bacteria, indicating that the biofilm is initially formed after only 4 days of immersion.

Unlike bare Ti, the general view of Ti–blank coating basically maintains a porous structure (Figure 11b-1) after 4 days of incubation. Numerous micro-sized rod-shaped features can be seen in Figure 11b-2, indicating extensive adhesion and proliferation of SRB on the Ti–blank coating. Figure 11b-3 shows the detailed morphological features of the SRB. It is clear that most of the SRB remain intact during pumping, indicating a limited capability of Ti–blank coating to kill SRB.

Figure 11c-1 exhibits the general view of the Ti–Cu coating after 4 days of incubation, displaying a porous morphology similar to that of the original coating (Figure 2). Figure 11c-2 shows a magnified view of the Ti–Cu coating, which shows a significant reduction in the number of SRB compared to the Ti–blank coating. Further magnification in Figure 11c-3 shows the appearance of bacterial lysis and also the disruption of the integrity of the bacterial membrane, demonstrating the antimicrobial capability of the Ti–Cu coating. Therefore, it is obvious that the Ti–Cu coating has a stronger antifouling capability compared to the Ti–blank coating and bare Ti substrate.

## 4. Discussion

The Ti–Cu coating fabricated via adding Na_2_Cu–EDTA to the electrolyte exhibits a distinctive microstructure (Figure 3, Figure 4, Figure 5, Figure 6, Figure 7 and Figure 8), a comparable corrosion resistance (Figure 9 and Figure 10), and an improved antifouling capability against SRB (Figure 11), relative to the Ti–blank coating.

During the MAO process, the voltage initially increases in an almost linear fashion then slows down after the dielectric breakdown and finally stabilizes (Figure 3) [44]. Initially, a gas envelope consisting mainly of oxygen is formed, which then reacts with Ti to form a thin titanium oxide film. As the applied voltage increases, the oxide film thickens. The growth of the MAO coating is mainly attributed to the electrochemical reaction to form TiO_2_ with limited incorporation of other species, especially a Cu species, considering the notably larger size of the Cu–EDTA complex relative to other negatively-charged species. When the voltage exceeds the spark voltage, local dielectric breakdown occurs, accompanied by micro-arc discharges. The formation of the discharge channel promotes the penetration of the electrolyte. In addition, the micro-arc discharge releases a large amount of thermal energy, which can lead to a significant increase in the local temperature and accelerate the plasma-activated electrochemical reaction.

The incorporation of Cu is believed to mainly occur after the dielectric breakdown of the as-formed coating when the applied voltage exceeds the breakdown potential. Under the promotion of an external electric field and mechanical convection, the Cu–EDTA complexes concentrate in the region immediately next to the anode. When the plasma discharge preferentially occurs at selected sites with relatively low dielectric constants, the thermal transient results in the melting of TiO_2_, which ejects outwards and then solidifies rapidly when exposed to the cool bulk electrolyte. During the solidification process, the Cu–EDTA complex may be captured by the molten TiO_2_. Since the thermal transient could significantly increase the local temperature, the captured Cu–EDTA complex fails to remain stable. Cu–EDTA complexes are first converted to copper-containing hydroxides, which are then thermally dehydrated to form copper oxides, as shown in the following reactions [40]:

[Cu–EDTA]^2−^ + nOH^−^ → Cu (OH)_n_ + EDTA^4−^ (2)

XCu (OH)_n_ → Cu_X_O + H_2_O (3)

When the above reactions proceed, the Cu species can be incorporated into the porous TiO_2_ (Figure 5 and Figure 6) and exist as Cu oxide, as suggested by the literature [43]. In contrast, EDTA was not noticed in the coating (Figure 5 and Figure 6), which could be associated with the high local temperature in the vicinity of a micro-arc discharge site that may promote its thermal decomposition.

After the ejection of TiO_2_ associated with the plasma discharge, a micro-sized crater, namely a discharge channel, could be formed (Figure 3), which promotes the mass transfer of Cu–EDTA complexes deep into the coating and further enhances the Cu incorporation into TiO_2_ (Figure 6). As a result, the relatively uniform distribution of Cu could be detected after adding Na2Cu–EDTA to the electrolyte, which forms a Cu-doped TiO_2_ coating, namely the Ti–Cu coating.

In addition to the chemical composition, the morphological features are also obvious between Ti–Cu and Ti–blank coatings. In the MAO process, the occurrence of plasma discharge promotes the ejection of molten TiO_2_ and results in the formation of micro-sized craters. Therefore, a porous appearance of the MAO coating could be revealed (Figure 3). EDTA species have a buffering effect [40]. The existence of EDTA species retards the structural destruction of the oxide layer and stabilizes the electrical double layer [45], which results in a sustainable plasma discharge with a relatively low energy density. In addition, the plasma discharge in the Ti–Cu coating may release a low level of energy relative to the Ti–blank coating since copper oxides exhibit relatively lower dielectric constants than TiO_2_ [40]. Hence, the addition of the Cu–EDTA complex to the electrolyte decreases the energy density of individual plasma discharge events, leading to a reduction in average pore size and porosity (Figure 4). Similarly, the thickening process may also be hindered by EDTA (Figure 6), which results in a decrease in the thickness of the Ti–Cu coating compared to the Ti–blank coating. It has even been reported that EDTA may enhance the inward growth of the MAO coating due to its strong ability to promote the anodic dissolution of the metallic Ti substrate in an aqueous solution [46]. Meanwhile, the thermal stress associated with the rapid increase and decline in local temperature may cause cracks in the MAO coating (Figure 3). The relatively low energy density with the presence of the Cu–EDTA complex could inhibit the thermal cracking to an extent, which thus leads to a less defective appearance relative to Ti–blank coating.

The phase component could also be affected by the addition of the Cu–EDTA species (Figure 7). The presence of EDTA inhibits the structural decomposition of the oxide layer, leading to a decrease in the energy density of individual plasma discharge events. Since the transition from metastable anatase to thermodynamically preferred rutile only occurs at an elevated temperature [47], the presence of the Cu–EDTA complex results in a reduced energy density and thus a reduction in rutile content. In addition, it is also noticed that only TiO_2_-related phases could be identified without the Cu-containing compounds, which may be due to the high temperatures in the MAO process leading to the melting of the Cu-compounds or the amorphous condition of those compounds.

Generally, a chemically inert ceramic coating possesses anticorrosion properties mainly due to its ability to inhibit electrolyte penetration into the coating/substrate interface [48,49,50]. Therefore, thickness and defects are critical to their ability to act as a physical barrier to electrolyte penetration. In this work, the Ti–Cu coating exhibits a reduced thickness compared to the Ti–blank coating (Figure 6). However, the reduction in the porosity, pore size, and cracking of the Ti–Cu coating relative to the Ti–blank coating contributes to its enhanced corrosion resistance (Figure 3 and Figure 4). In addition, copper oxides typically have reduced electrical conductivity compared to TiO_2_, which thus retards the electrical transportation through TiO_2_. Hence, the incorporation of Cu into a TiO_2_ coating remains harmless to its corrosion resistance (Figure 9 and Figure 10).

Finally, it is clear that the incorporation of Cu enhances the antifouling ability of TiO_2_ against SRB (Figure 11), which is closely related to their different surface chemical properties that affect SRB proliferation on the surface of the coating. After the adhesion of SRB, the surface chemistry may interrupt its proliferation due to the antimicrobial ability of the Cu species [51]. Since no released Cu ions were detected by inductively coupled plasma (ICP) after the immersion in culture media for 4 days, it was concluded that the Cu species in TiO_2_ acts as an effective antibacterial agent to hinder the proliferation of SRB.

Cu species have been proven to promote the production of reactive oxygen species (ROS), which may lead to an abrupt decrease in cell membrane integrity [52]. Meanwhile, Cu species also have a strong affinity with intracellular proteins, which could inactivate the vital enzyme and DNA in bacteria and finally disable their replication capability when SRB adhere to the Cu-containing surface [53]. Hence, Cu oxides contribute to the bactericidal effect of SRB and inhibit their growth. As a result, the Ti–Cu coating provides better antifouling compared to the Ti–blank coating and the titanium metal substrate.

## 5. Conclusions

In this study, the microstructure of copper-doped titanium dioxide coatings and its correlation with properties were systematically investigated, and the conclusions are as follows:

➢ Cu-doped TiO_2_ coatings can be generated on Ti substrates after an MAO treatment in a solution containing Na_2_Cu–EDTA.
➢ Relative to the Cu-free coatings, the Cu-doped TiO_2_ coatings showed fewer defects with a reduced ratio between rutile and anatase.
➢ The formation of TiO_2_ coating improves the corrosion resistance of the Ti substrate, while copper incorporation is insensitive to the effect of corrosion resistance.
➢ The incorporated Cu is responsible for the enhanced antifouling performance of the Cu-doped TiO_2_ coating compared to the Cu-free coating and Ti substrates.

## Figures and Tables

**Table 1:** Quantitative element (wt. %) compositions of MAO coatings.

	Ti	O	Ca	P	Cu
Ti–blank	53.99	37.71	4.79	3.51	0
Ti–Cu	53.31	36.88	3.80	4.28	1.73

**Table 2:** Tafel slopes (β_c_), corrosion current densities (*i_corr_*), and corrosion potentials (*E_corr_*).

	*β*_c_ (mV)	*I*_corr_ (A·cm^−2^)	*E*_corr_ (mV)
Bare Ti	82.38	2.10 × 10^−7^	−350
Ti–blank	58.85	1.64 × 10^−8^	100
Ti–Cu	49.36	1.65 × 10^−8^	180

**Table 3:** Fitting data of EIS of MAO coatings.

Parameter	Bare Ti	Ti–Blank	Ti–Cu
*R*_s_ (Ω·cm^2^)	5.02	6.81	8.78
*CPE*_1_ (F cm^−2^)	8.71 × 10^−5^	5.46 × 10^−6^	4.37 × 10^−6^
*n-CPE*_coat_	0.89	0.71	0.72
*R*_1_ (Ω·cm^2^)	617.7	627.3	444.2
*CPE*_dl_ (F cm^−2^)	3.04 × 10^−4^	4.07 × 10^−5^	2.61 × 10^−5^
*n-CPE*_dl_	0.73	0.77	0.75
*R*_ct_ (Ω·cm^−2^)	1.08 × 10^4^	1.85 × 10^5^	2.65 × 10^5^

## Data Availability

Data presented in this article are available upon request from the corresponding author.
